# THE CHALLENGE OF ASSESSING AND MEASURING SOCIAL INCLUSION AND EXCLUSION IN LATER LIFE

**DOI:** 10.1093/geroni/igac059.468

**Published:** 2022-12-20

**Authors:** Charles Waldegrave, Marja Aartsen

## Abstract

Social exclusion is a serious problem that can lead to diminished well-being, health problems, premature death and increased societal costs. Depending on the definition used, 10 to 30% of the older adults experience social exclusion, and many have been confronted with prolonged isolation during the pandemic. Constructing measures for social inclusion and exclusion is a challenging yet important endeavor, and various approaches exist. For example, EU-policy makers define social exclusion mainly in terms of poverty and lack of labor market participation However, a too narrow definition of social exclusion leaves large groups of people unattended leading to sub-optimal understandings of social exclusion and ineffective interventions. This symposium brings together scholars from different cultures. The first paper discusses newly developed social and well-being scales, that more adequately address cultural notions of exclusion and discrimination experienced by older Māori (indigenous New Zealanders). Based on unique data from time-diaries kept by older women from several European countries and the U.S, the second speaker discusses how increased time spend alone is key factor behind widows’ reduced well-being. The last study finds a northwest to southeast gradient in objective exclusionary states, with the rates in southeast Europe to be pronounced among older women.

